# A Systematic Evaluation of Supervised Machine Learning Algorithms for Cell Phenotype Classification Using Single-Cell RNA Sequencing Data

**DOI:** 10.3389/fgene.2022.836798

**Published:** 2022-02-23

**Authors:** Xiaowen Cao, Li Xing, Elham Majd, Hua He, Junhua Gu, Xuekui Zhang

**Affiliations:** ^1^ School of Artificial Intelligence, Hebei University of Technology, Tianjin, China; ^2^ Department of Mathematics and Statistics, University of Victoria, Victoria, BC, Canada; ^3^ Department of Mathematics and Statistics, University of Saskatchewan, Saskatoon, SK, Canada; ^4^ School of Science, Hebei University of Technology, Tianjin, China

**Keywords:** classification, gene selection, ensemble algorithms, machine learning, single-cell RNA sequencing, supervised algorithms

## Abstract

The new technology of single-cell RNA sequencing (scRNA-seq) can yield valuable insights into gene expression and give critical information about the cellular compositions of complex tissues. In recent years, vast numbers of scRNA-seq datasets have been generated and made publicly available, and this has enabled researchers to train supervised machine learning models for predicting or classifying various cell-level phenotypes. This has led to the development of many new methods for analyzing scRNA-seq data. Despite the popularity of such applications, there has as yet been no systematic investigation of the performance of these supervised algorithms using predictors from various sizes of scRNA-seq datasets. In this study, 13 popular supervised machine learning algorithms for cell phenotype classification were evaluated using published real and simulated datasets with diverse cell sizes. This benchmark comprises two parts. In the first, real datasets were used to assess the computing speed and cell phenotype classification performance of popular supervised algorithms. The classification performances were evaluated using the area under the receiver operating characteristic curve, F1-score, Precision, Recall, and false-positive rate. In the second part, we evaluated gene-selection performance using published simulated datasets with a known list of real genes. The results showed that ElasticNet with interactions performed the best for small and medium-sized datasets. The NaiveBayes classifier was found to be another appropriate method for medium-sized datasets. With large datasets, the performance of the XGBoost algorithm was found to be excellent. Ensemble algorithms were not found to be significantly superior to individual machine learning methods. Including interactions in the ElasticNet algorithm caused a significant performance improvement for small datasets. The linear discriminant analysis algorithm was found to be the best choice when speed is critical; it is the fastest method, it can scale to handle large sample sizes, and its performance is not much worse than the top performers.

## 1 Introduction

Single-cell RNA-sequencing (scRNA-seq) technology enables researchers to investigate a genome at the single-cell level with high resolution, and it was named Method of the Year 2013 (Editorial, 2014). The analysis of scRNA-seq data has played a vital role in understanding intrinsic and extrinsic cellular processes in biological and biomedical research (Wang et al., 2019). The scRNA-seq technique has provided us with an opportunity to identify the cellular compositions of complex tissues (Svensson et al., 2018; Cheng et al., 2020), which is valuable for detecting new populations of cells, defining different cell types, and discovering rare cells that represent minor cell types. Hence, one of the most popular tasks in the analysis of scRNA-seq data is the classification of various cell-level phenotypes, such as cell-type annotation. Pasquini et al. (2021) discussed 24 recently developed cell-type annotation methods based on unsupervised and supervised machine learning algorithms. The majority of these methods are based on supervised machine learning algorithms, especially the newer ones. The standard annotation approach is clustering followed by manual annotation of each cluster. However, this not only is tedious and inefficient (Qi et al., 2020) but also produces inconsistent results due to the subjective human decisions made in the process of annotation. A large quantity of scRNA-seq data has been generated and made publicly available recently, and this means that training supervised machine learning models is now a preferred approach.

Since many supervised machine learning algorithms can be (and have been) applied to the popular task of cell phenotype classification, we are interested in evaluating their performance under various conditions. Abdelaal et al. (2019) conducted a benchmark study to compare currently available software for cell identification, and this serves as a great resource to help users to select a software package for their analyses. Each software package has an underlying machine learning algorithm and specific add-on components or data-analysis steps, and most add-ons can be applied to other methods. Developers are mostly interested in which underlying machine learning algorithm is most suitable for scRNA-seq data analysis and should hence be used to develop their next software release. This motivated us to conduct a benchmark study to conduct a fair comparison of the currently used supervised machine learning algorithms without any add-ons.

The characteristics of a dataset can have a critical effect on the performance of a machine learning method. A standard scRNA-seq training dataset for cell phenotype classification consists of a genomic data matrix and class label for each cell. In the genomic data matrix, each record (row) represents a cell and each column represents a single gene’s expression level; there are tens of thousands of genes with a very complex correlation structure. The correlation structure varies between datasets, making it necessary to evaluate the performance of methods using several datasets. Each cell in the data matrix is the gene expression level, or the count of RNA fragments mapped to a given gene. Due to the limitations of the technology (dropout), there is a large proportion of zeros in these counts. However, their corresponding actual expression levels are not zero. The zero-inflated negative binomial distribution is the most popular distribution used to model expression levels in scRNA-seq data. However, a recent benchmark study showed that such a distribution does not have a clear advantage in differential expression analysis (Soneson and Robinson, 2018).

In short, most genomic data matrices share the same characteristics, as discussed above. The only key factor that could affect classification performance is the sample size of the dataset. Therefore, in our benchmark study, we investigated data of various sample sizes. First, following the protocol described for a previous benchmark study (Soneson and Robinson, 2018), we selected 27 datasets from the real datasets of Conquer. We considered these datasets as small-sample-size data, since more recent scRNA-seq datasets have become much larger than that used in 2018. Hence, we used datasets with larger sample sizes: four medium-sized datasets and 12 large ones (Packer et al., 2019). Details of the benchmark data can be found in Supplementary Tables S1–S3.

In this study, we considered eight individual algorithms used in software for cell phenotype classification: 1) the ElasticNet algorithm (Zou and Hastie, 2005), which is used in the Garnett software package (Pliner et al., 2019); 2) ElasticNet with interactions, to learn whether adding interaction terms improves classification performance; 3) the linear discriminant analysis (LDA) algorithm (Xanthopoulos et al., 2013), which is used in the R library scID (Boufea et al., 2020); 4) NaiveBayes (NB) (John and Langley, 2013), which is used in CellO (Bernstein and Dewey, 2021); 5) the support vector machine (SVM) algorithm (Steinwart and Christmann, 2008), which is used in scPred (Alquicira-Hernandez et al., 2019); 6) the K-nearest neighbors (KNN) algorithm (Kramer, 2013), which is used in scANVI package (Xu et al., 2021) and the scClassify package (Lin et al., 2020); 7) the Tree algorithm (Gupta et al., 2012), which is used in the CHETAH package (de Kanter et al., 2019); and 8) the XGBoost (XGB) algorithm (Friedman and Meulman, 2003), which is used in the CaSTLe package (Lieberman et al., 2018). Ensemble learning algorithms usually work better than a specific individual algorithm (Dietterich, 2002). Therefore, we also constructed five ensemble algorithms based on the weighted votes of the eight individual algorithms discussed above; we then evaluated their performance against the individual algorithms.

Using all of the datasets, we compared the classification performance and running times of these methods. We found that ElasticNet with interaction terms had the best classification performance when the sample size was not large, and XGB worked best with large datasets. The LDA algorithm had reasonable classification performance and ran the fastest in most cases. When the sample size is large, ElasticNet takes a very long time to run. The NB algorithm also had great performance in the medium-sized datasets we investigated. We also evaluated the performance of these algorithms for gene selection. Such evaluation requires a list of “true” genes, so only simulated datasets were used in this part. We found that ElasticNet had the best performance in gene selection.

## 2 Materials and Methods

This section describes the datasets, algorithm design, evaluation criteria, classification methods, and gene selection.

### 2.1 Datasets

The datasets from Conquer (Soneson and Robinson, 2018) and GSE126954 (Packer et al., 2019) were used to evaluate all classification methods for scRNA-seq data. A total of 27 datasets from Conquer were applied as small datasets, four datasets from GSE126954 were used as medium-sized datasets, and 12 datasets from GSE126954 were considered as large ones (Supplementary Tables S1–S3). Finally, as an additional type of dataset, the simulated datasets provided by Soneson and Robinson (2018) were used to evaluate gene-selection performance. For the details of these simulated datasets, see Supplementary Table S4.

#### 2.1.1 The 27 Datasets From Conquer

The Conquer repository (Soneson and Robinson, 2018) was developed at the University of Zurich, Switzerland. The datasets used in this research were downloaded from http://imlspenticton.uzh.ch:3838/conquer. Although Conquer contains 40 scRNA-seq datasets, we selected 27 of these with two types of cells in a specific phenotype, and there were at least 15 cells of each type (please refer to Supplementary Table S1). The predictors were carefully chosen from the top 1,000 genes with the strongest correlations. We named these datasets “small datasets.”

#### 2.1.2 GSE126954 Datasets

The GSE126954 datasets were presented by Packer et al. (2019), and they can be downloaded from https://www.ncbi.nlm.nih.gov/geo/query/acc.cgi?acc=GSE126954. These datasets involve 86,024 single-cell scRNA-seq sets from *Caenorhabditis elegans* embryos (Packer et al., 2019). We divided these into two parts. The first part contained four datasets that were larger than the small datasets from Conquer; we selected four pairs of different cells to form four “medium-sized datasets.” The number of cells in our selected datasets ranged from 112 to 911 (please refer to Supplementary Table S2 for more details). The second part contained 12 datasets named “large datasets.” Additionally, this paper used two datasets based on different cell types and ten datasets according to a pairwise comparison of scRNA-seq profiles of cells from *C. elegans* embryos at varying developmental stages. The numbers of each type of cell ranged from 1,732 to 25,875, as described in Supplementary Table S3. These two parts of the datasets were used to choose 1,000 genes with the strongest correlations as predictors. The algorithm parameter settings were the same as those used for the small datasets.

#### 2.1.3 Simulated Conquer Datasets

To evaluate feature selection, it is necessary to know the identity of the “real” gene. In this study, we used three simulated datasets provided by Soneson and Robinson (2018) and Soneson and Robinson (2016). The simulations were conducted using the *powsimR* package (Vieth et al., 2017). The simulation input parameters were learned from the three real datasets: GSE45719, GSE74596, and GSE60749-GPL13112. We used information about the real class members of each cell received from the real datasets. The simulated and real datasets can be freely downloaded (Soneson and Robinson, 2016). Table 1 presents more information about these simulated datasets.

**TABLE 1:** Computation times of each algorithm with each dataset. Each column represents a different algorithm, and the rows indicate different datasets. The smallest computation times are illustrated by underlining.

Label	ElasticNet	ElasticNet with interactions	LDA	NB	SVM	KNN	Tree	XGB	Sample size
The computation time in 27 small datasets (seconds)									
1	37.057	60.918	1.969	7.136	639.367	9.681	15.915	29.133	192
2	14.362	24.385	0.980	5.307	247.713	3.990	8.028	12.114	95
3	198.204	30.126	5.943	14.138	5,208.412	68.672	35.271	107.450	564
4	24.998	38.792	0.996	5.723	290.221	4.500	9.270	13.388	110
5	40.536	65.178	1.682	7.300	632.190	9.118	11.990	22.355	192
6	38.661	64.686	1.626	7.203	615.368	8.911	10.710	19.079	186
7	20.004	29.290	0.877	5.219	240.178	3.889	9.563	13.565	99
8	40.087	61.180	1.661	6.916	650.740	9.053	14.454	26.990	192
9	37.518	35.188	1.494	6.675	411.244	7.798	10.358	18.767	91
10	80.411	121.676	2.319	8.260	835.417	15.678	12.739	25.214	268
11	37.893	58.476	1.283	6.141	339.512	6.335	10.064	16.080	147
12	430.164	170.021	9.560	18.190	10 ,328.148	161.913	18.037	121.704	192
13	104.301	111.083	1.631	7.095	673.008	8.861	10.582	34.456	192
14	100.775	111.154	2.659	9.619	1,545.478	22.382	14.804	37.964	328
15	116.645	125.838	3.809	11.827	3,184.677	46.696	26.341	58.991	460
16	56.745	65.334	1.440	7.215	553.960	9.138	11.650	21.656	183
17	353.498	33.495	7.221	16.828	1,288.900	126.284	34.363	90.167	106
18	96.279	101.553	2.455	9.689	1,539.189	21.260	19.310	37.981	313
19	35.485	25.004	0.897	6.041	277.117	4.730	9.726	15.810	91
20	25.816	27.386	0.760	5.652	238.028	4.020	7.990	12.259	96
21	178.537	164.780	1.456	7.561	440.966	9.325	8.794	19.780	188
22	43.940	50.828	1.403	7.359	566.504	9.146	10.180	18.819	181
23	72.664	83.030	1.502	7.613	657.644	9.504	9.713	27.458	192
24	66.112	96.200	1.487	7.616	639.454	9.488	10.289	27.005	192
25	59.439	86.527	1.528	7.638	664.293	9.681	13.983	29.074	192
26	126.590	85.750	5.288	14.354	3,649.444	74.889	33.485	78.146	269
27	30.640	53.641	1.339	7.032	481.385	7.598	12.044	20.131	164
The computation time in 4 medium datasets (seconds)									
1	163.504	236.97	11.765	23.030	4,194.281	176.300	32.935	90.274	843
2	233.984	670.67	16.954	29.639	9,350.588	325.453	55.913	139.470	1,126
3	640.719	46.010	14.902	22.276	12 ,406.980	263.876	48.200	121.668	992
4	41.442	164.89	2.660	10.531	518.837	22.619	14.459	27.354	234
The computation time in 12 large datasets (seconds)									
1	485.537	NA	247.775	391.010	NA	NA	724.572	2,566.135	19 ,252
2	258.447	NA	249.383	400.039	NA	NA	676.196	1,792.239	5,328
3	2,276.346	NA	282.598	380.155	NA	NA	920.946	1,910.279	21 ,492
4	1,932.343	NA	235.466	405.532	NA	NA	626.151	2,986.626	22 ,661
5	9,093.946	NA	165.728	267.974	NA	NA	357.760	1,150.460	23,718
6	9,026.280	NA	510.495	741.520	NA	NA	1,469.216	7,039.001	20 ,213
7	31,978.390	NA	370.260	492.451	NA	NA	913.547	3,274.896	13 ,577
8	7,256.621	NA	346.646	520.316	NA	NA	923.122	2,940.168	33 ,043
9	10 ,807.530	NA	466.657	667.569	NA	NA	1,163.817	3,267.698	27 ,700
10	1,961.241	NA	384.110	551.439	NA	NA	1,242.170	3,313.920	28 ,757
11	11 ,386.465	NA	233.273	434.554	NA	NA	829.204	2,607.679	36 ,407
12	2,691.988	NA	65.735	116.332	NA	NA	231.935	792.720	37 ,464

### 2.2 Classification Methods

We compared the results of ElasticNet, ElasticNet with interactions, LDA, NB, SVM, KNN, Tree, XGB, and five ensemble algorithms. The first eight methods repeated 100 rounds of tenfold cross-validation. The grouping of each round of cross-validation was random. The results of the classifications by the five ensemble algorithms were obtained from seven traditional algorithms: ElasticNet, LDA, NB, SVM, KNN, Tree, and XGB. We calculated the area under the receiver operating characteristic curve (AUC), F1-score, false-positive rate (FPR), Precision, and Recall of each algorithm. We also compared the computation time of each algorithm.

#### 2.2.1 ElasticNet

As a combination of ridge regression and lasso regression, ElasticNet can not only reduce the prediction variance but also achieve coefficient shrinkage and variable selection (Soomro et al., 2016; Lu et al., 2021). This algorithm involves two parameters, *α* and *λ*:

$$\lambda \overset{p}{\underset{j=1}{\sum }}\left(\alpha \beta_{j}^{2}+\left(1-\alpha \right)\left|\beta_{j}\right|\right)$$ (1)

To evaluate *α*, we selected six values distributed evenly between 0 and 1 $\left\{0,0.2,0.4,0.6,0.8,1\right\}$ , and for evaluation of *λ*, we chose 100 values from log  10^–8^ to   log  10^5^. Note that in the 12 large datasets, the parameters *α* and *λ* were changed and set using the full data to avoid the computation taking an excessively long time. The ElasticNet model was also applied to the complete dataset to select the best *α* and *λ* values from the 12 datasets, and these were then directly used in the tenfold cross-validation experiments. In the small and medium-sized datasets, we also considered ElasticNet with 200 interactions. We combined 1,000 genes in pairs to form interactions and then applied logistic regression to find the 200 interactions with the strongest correlations with the response variable.

#### 2.2.2 Linear Discriminant Analysis

The LDA technique identifies a linear combination of predictors that maximize the between-class scatter and minimize the within-class scatter (Park and Park, 2008). It uses the label information to learn a discriminant projection that can enlarge the between-class distance and reduce the within-class distance to improve the classification performance. Various extensions of LDA have been developed to enhance its performance and efficiency (Tharwat et al., 2017). In this benchmark study, we used the LDA function in the R package MASS with parameters set to their default values.

#### 2.2.3 NaiveBayes

The NB classifier defines the probability of an item belonging to a particular class. It is based on Bayes’ theorem with the assumption that the features are independent. However, this assumption may cause a problem because real-world features are generally interdependent (Malik and Kumar, 2018). We used the naiveBayes function in the e1071R package with the value of Laplace set to 1.

#### 2.2.4 Support Vector Machine

SVMs are formulated for two-class single-label problems (Hasan et al., 2013). The appropriate kernel and its parameters selected for a specific classification problem can influence the performance of an SVM (Hasan et al., 2017). We adopted tenfold cross-validation to select an optimal value for *γ*, which is a parameter that needs to be defined when the radial basis function is chosen as the kernel. This implicitly determines the distribution of the data mapped to the new feature space. The larger the value of *γ*, the smaller the number of support vectors; the smaller the value of *γ*, the larger the number of support vectors. The number of support vectors affects the speed of training and prediction. Another parameter is the cost; this is a penalty coefficient that defines the tolerance for errors. The higher the cost, the smaller the error tolerance; it is easy to overfit with higher cost values and easy to underfit with lower cost values. As such, the generalization ability of the model will become poor if the cost is either too high or too low. The range of *γ* was $\left\{\frac{\left(\frac{1}{n}\right)}{10},\left(\frac{1}{n}\right),\left(\frac{1}{n}\right)\times 10\right\}$ , where *n* represents the number of genes. The range of the cost is $\left\{0.01,0.1,1,10,100\right\}$ . Predictions were then made with the help of the optimal model.

#### 2.2.5 K-Nearest Neighbors

The principle of the KNN algorithm is that if most of the *k* most-similar samples to a query point *q*_*i*_ in the feature space belong to a particular category, then an inference can be made that the query point *q*_*i*_ also falls into this category. The distance in the feature space can measure similarity, so this algorithm is called the KNN algorithm. A training dataset with accurate classification labels should be known at the beginning of the algorithm. Then, for a query data point *q*_*i*_ whose label is not known and which is represented by a vector in feature space, the distances between it and every point in the training dataset are calculated. After sorting the results of this distance calculation, a class label for the test point *q*_*i*_ can be applied according to the labels of the *k* nearest points in the training dataset (Kuang and Lei, 2009). When the sizes of the training and test datasets are both very large, the execution time of these distance calculations may be the bottleneck of the application of the KNN algorithm (Kuang and Lei, 2009).

#### 2.2.6 Tree

The Tree algorithm is a hierarchical structure in which internal tree nodes represent splits applied to decompose the domain into regions, and terminal nodes assign class labels or class probabilities to regions believed to be sufficiently small or sufficiently uniform (Kozdrowski et al., 2021). In this work, pruning was carried out using tenfold cross-validation. To avoid the situation in which only one branch was left after pruning—which would lead to unpredictable classification results—pruning was not allowed if the leaf size after pruning would have been less than two.

#### 2.2.7 XGBoost

XGBoost is a regression tree that has the same decision rules as a standard decision tree; it supports both regression and classification. This algorithm is an efficient and scalable variant of the gradient boosting machine (Ma et al., 2020). The XGB method can handle sparse data and can thus be implemented flexibly using distributed or parallel computing (Wang et al., 2017; Chang et al., 2018).

#### 2.2.8 Five Ensemble Algorithms

Ensembles are achieved by generating different algorithms and combining their results into a single consensus solution (Chiu and Talhouk, 2018). This study used five ensemble algorithms constructed from the basic results of algorithm predictions applied to classification. In this work, two of the ensemble algorithms used soft decision rules and the other three used hard decision rules.

We define *p*_*n*,*i*_ as the predicted probability of the *n*th sample from the *i*th algorithm, where *i* is the index of the algorithm from \(ElasticNet, LDA, NB, SVM, KNN, Tree, XGB\). The soft ensemble rules made decisions based on the weighted average of the predicted probabilities from all methods:

$${\tilde{p}}_{n}=\frac{\sum_{i}p_{n,i}\times w_{i}}{\sum_{i}w_{i}},$$ (2)

where *w*_*i*_ represents the classification performance of each method. This study used two criteria for *w*_*i*_—F1-Median and AUC-Median—to construct two ensemble approaches. The ensemble methods classified the *n*th sample by the weighted probability ${\tilde{p}}_{n}$ . We named the two ensemble algorithms “ensemble-weighted.AUC” and “ensemble-weighted.F1.”

We denote *O*_*n*,*i*_ as the predicted class (0 or 1) of the *n*th sample from the *i*th algorithm. In the hard ensemble rules, a decision was made based on the weighted votes from all methods:

$${\tilde{O}}_{n}=\left\{\begin{array}{c} 0;\;\text{if}\;\underset{\left\{i:{\text{O}}_{n,i}=0\right\}}{\sum }w_{i}\geq \underset{\left\{i:{\text{O}}_{n,i}=1\right\}}{\sum }w_{i},\;\\ 1;\;\text{if}\;\underset{\left\{i:{\text{O}}_{n,i}=0\right\}}{\sum }w_{i}<\underset{\left\{i:{\text{O}}_{n,i}=1\right\}}{\sum }w_{i},\; \end{array}\right.$$ (3)

where *w*_*i*_ is constant 1 or AUC-Median or F1-Median; indeed, *w*_*i*_ was used to construct three hard ensemble rules. We named these three ensemble algorithms “ensemble-vote,” “ensemble-addition.AUC,” and “ensemble-addition.F1.”

### 2.3 Design of Evaluation Experiments

#### 2.3.1 Cross-Validation

To evaluate the classification performance of the supervised algorithms, we carried out tenfold cross-validation in 100 rounds after filtering the genes, phenotypes, and cells. The complete set of samples, which were labeled as either 0 or 1, were divided into ten groups in each round. In each of the 100 rounds of cross-validation, we applied a confusion matrix for either cell phenotype classification or gene selection. From these confusion matrices, we calculated the following parameters to compare the performance of the methods: AUC, F1-score, FPR, Precision, and Recall.

#### 2.3.2 Evaluation of Classification

For each dataset, each sample result in 100 rounds of experiments was predicted to be either 0 or 1 (with a threshold of 0.5 dividing the predicted results into 0 and 1). The confusion matrix was constructed according to the prediction results and actual values from each algorithm. The numbers of samples were achieved with both real and predicted values of 0 (*a*), actual values of 0 and predicted values of 1 (*b*), actual values of 1 and predicted values of 0 (*c*), and both actual and predicted values of 1 (*d*). “Recall” measures the correct ratio in samples with an actual value of category 1; the calculation formula for this is *d*/(*c* + *d*) (Hand, 2009). “Precision” measures the ratio of samples with an actual value of 1 where it should be 1; the formula for this is *d*/(*b* + *d*). The calculation formula for the F1-score is (2 ×Recall ×Precision)/(Recall + Precision). The FPR measures the proportion of samples with an actual value of 0 but a predicted value of 1; the calculation formula for this is *b*/(*a* + *b*). As noted earlier, AUC measures the area under the receiver operating characteristic curve.

#### 2.3.3 Evaluation for Computation Time

To benchmark each algorithm according to the computation time taken for each dataset, we recorded the computation time of each algorithm for 100 rounds and calculated the average values.

#### 2.3.4 Evaluation of Gene Selection

The algorithms considered were NB, Tree, XGB, and ElasticNet. We selected the 5,000 genes from the three simulated datasets provided by Soneson and Robinson (2018) and Soneson and Robinson (2016) that had the strongest correlations with the response variable. The system randomly selected 70*%* of the data from three simulated datasets as a training set and repeated this random selection 100 times. Then, gene selection was conducted on 100 subsets with 5,000 genes. A comparison was carried out between the selected genes and the real genes in the real datasets. We used a two-column matrix to help the calculation of each indicator; the first column contained the selected genes, and the second column contained the real genes. Finally, five criteria—AUC, F1-score, FPR, Precision, and Recall—were considered to evaluate the performance of the algorithms for gene selection.

For the Tree algorithm, we carried out cross-validation to prune the tree with minimum deviance. With the NB algorithm, we applied *recursive feature elimination* (Chen et al., 2020) to implement gene selection. The parameter settings of the other algorithms were the same as those used for classification.

## 3 Results

In this section, we describe the method comparison results in detail using each of the benchmark criteria. We summarize the classification performance and computation time for all datasets with various sample sizes. We then summarize the gene-selection performance using simulated datasets.

### 3.1 Classification Performance

As described, in this study, the performance of different classification algorithms on three types of datasets of different of sizes was examined. In this section, the results of each classification criterion are shown to compare the performance of the methods in the subsequent subsections.

**FIGURE 1:**
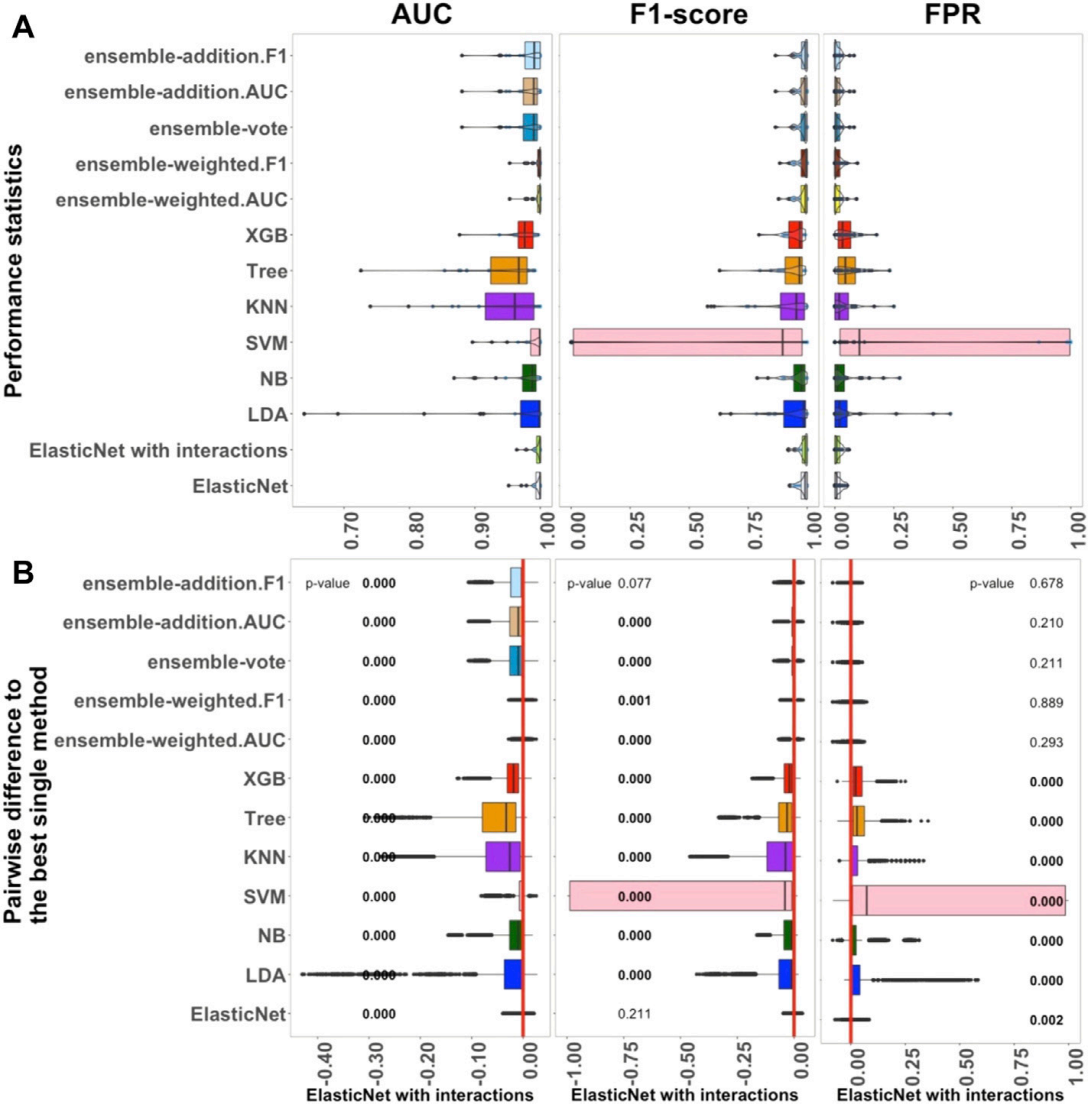
Performance of 13 algorithms with the 27 small datasets. The values of three criteria (AUC, F1-score, and FPR from left to right) are shown in **(A)**. There are 2,700 points in each box. The values of three criteria are used to represent the performance of the corresponding algorithm. **(B)** Differences between the best single algorithm and the other algorithms. From left to right, the results show the differences in AUC, F1, and FPR including the *p*-values from the Wilcoxon test; *p*-values <0.05 are indicated in bold, and the red lines indicate the zero value. The best single method is shown at the bottom of each box.

**FIGURE 2:**
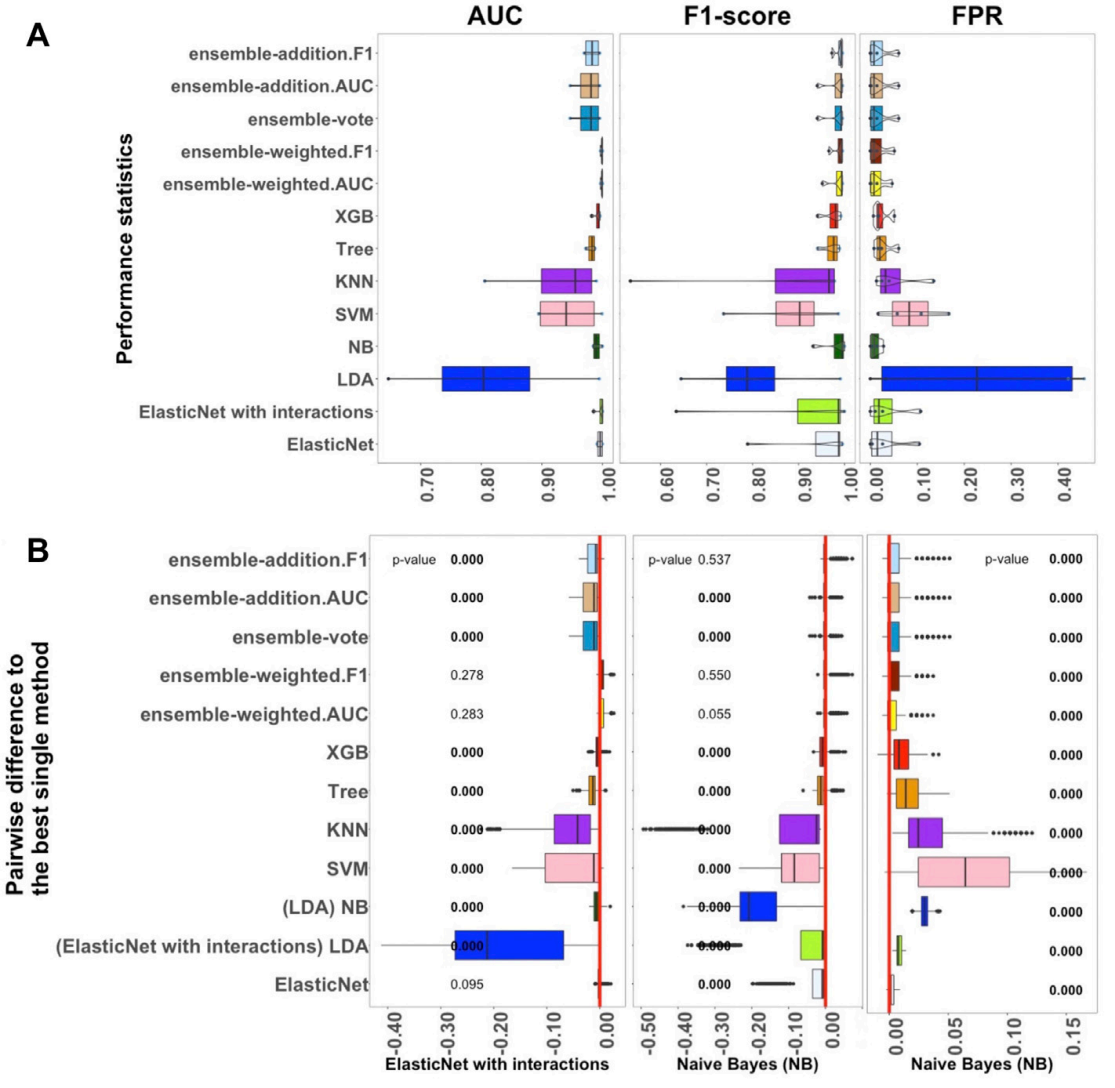
Performance of 13 algorithms with the four medium-sized datasets. The values of three criteria (AUC, F1-score, and FPR from left to right) are shown in **(A)**. There are 400 points in each box. The values of these three criteria were used to represent the performance of the corresponding algorithm. **(B)** Differences between the best single algorithm and the other algorithms. From left to right, the results show the differences in AUC, F1, and FPR including the *p*-values from the Wilcoxon test; *p*-values <0.05 are indicated in bold, and the red lines indicate the zero value. The best single methods, of which there were two, are shown at the bottom of the boxes.

**FIGURE 3:**
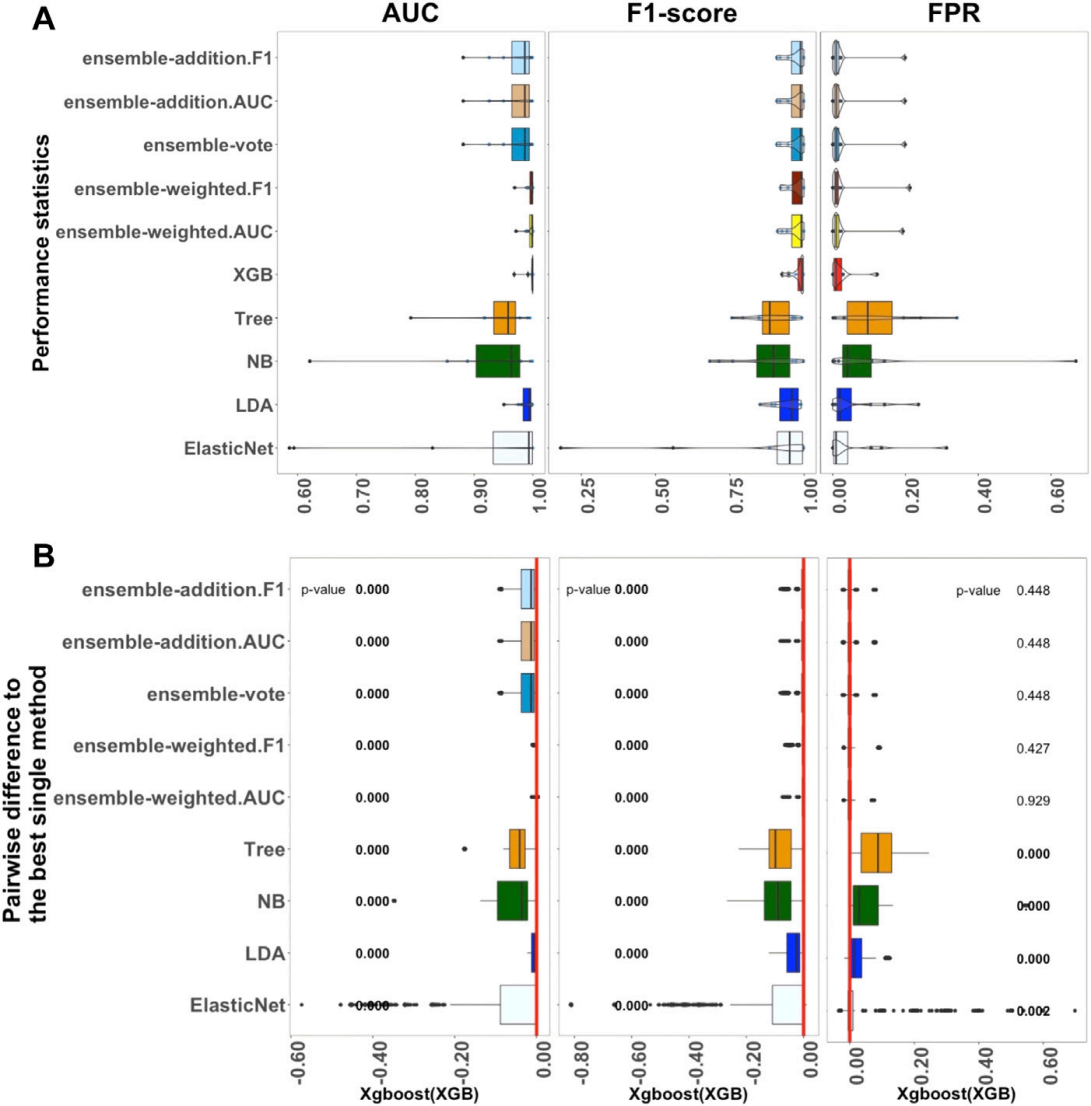
Performance of ten algorithms with the 12 large datasets. The values of three criteria (AUC, F1-score, and FPR from left to right) are shown in **(A)**. There are 1,200 points in each box. The values of three criteria were used to represent the performance of the corresponding algorithm. **(B)** Differences between the best single algorithm and the other algorithms. From left to right, the results show the differences in AUC, F1, and FPR including the *p* values from the Wilcoxon test; *p* values <0.05 are indicated in bold, and the red lines indicate the zero value. The best single method is shown at the bottom of each box.

Figures 1–3 show the results from the small, medium, and large datasets, respectively. In each of these figures: as three criteria for investigating the performance of the algorithms, panel A displays, from left to right, the AUC, F1-score, and FPR values of the classification outcomes; panel B shows the differences between the best single algorithm and the other algorithms. From left to right, the results in these panels display the *p*-values of the AUC, F1-score, and FPR. The red lines indicate the zero value of differences among the performances of algorithms. To closely examine these differences, the discrepancies between the performance of the best single algorithm and the other algorithms were investigated using an adjusted *p*-value cutoff of 0.05. The *p*-value was considered to three decimal places. Values below 0.05 are indicated in bold to highlight the significant differences.

The interquartile range (IQR) and median values (calculated from the results of 100 experiments for each dataset) for the five criteria are also given in Supplementary Figures S1–S3. In these figures, the first column shows the performance of the algorithms according to five criteria, AUC, F1-score, FPR, Recall, and Precision. The second column shows the AUC-Median, F1-Median, FPR-Median, Recall-Median, and Precision-Median. The third column shows the AUC-IQR, F1-IQR, FPR-IQR, Recall-IQR, and Precision-IQR, from top to bottom. The IQR values were used to represent the stability of the corresponding algorithm. Supplementary Figure S1 illustrates the performance of the 27 small datasets, Supplementary Figure S2 shows the result of the four medium-sized datasets, and Supplementary Figure S3 shows the results from the 12 large datasets.

#### 3.1.1 Benchmarks for Each Classifier in Small Datasets

The performance of 13 supervised algorithms was analyzed using 27 real datasets with relatively small sample sizes from the Conquer study (Soneson and Robinson, 2018), which has been used for intra-dataset evaluation. The studied datasets are relatively typically sized scRNA-seq datasets with 24 balanced datasets and three unbalanced datasets (Supplementary Table S1). The performance of the 13 algorithms (ElasticNet with and without interactions, LDA, NB, SVM, KNN, Tree, XGB, and five ensemble algorithms) considering AUC, F1-score, and FPR are shown in Figure 1. In Figure 1A, each box contains 2,700 scores, and each of these scores represents a single value from each experiment with each dataset. Overall, Figure 1 indicates that ElasticNet with interactions has better performance as a linear algorithm when compared to the others in terms of AUC, F1-score, and FPR. Considering Supplementary Figure S1, the Recall of ElasticNet was also considerably better than other non-ensemble algorithms. However, the five ensemble algorithms had higher precision than the non-ensemble algorithms (Supplementary Figure S1).

As shown in Figure 1A, from the linear algorithms, the median AUC values for ElasticNet with and without interactions, LDA, NB, and SVM, were greater than 0.987. For the 27 small datasets, the median AUC value of the non-linear Tree algorithm was 0.967, and it was 0.979 for the XGB; this was higher than the value for the KNN algorithm, 0.966. Thus, in small datasets, when AUC is considered as the main criterion, the linear algorithms performed better than the non-linear ones.

We also used each criterion’s IQR value to indicate the stability of the algorithms. Details of these results are shown in Supplementary Figure S1. The XGB algorithm was better than Tree in terms of both performance and stability. Among the five ensemble algorithms, it seemed that weighting by AUC and F1-score played an important role in improving the results. The median AUC values for the ensemble algorithms were approximately the same, each being over 0.990. According to the outcomes, in the small datasets, ElasticNet with interactions was selected as the best single method because it had the highest AUC and lower AUC-IQR values. It also had the best performance considering F1-score and FPR.

Figure 1B shows a pairwise comparison between the classification algorithms and the best selected single method (ElasticNet with interactions) under each criterion. There were significant differences among the performances of the algorithms, and these further reinforced the selection of ElasticNet as the best algorithm. Moreover, considering the AUC and F1-score criteria, the inclusion of interactions significantly improved the performance of ElasticNet. Among the single methods, the LDA, NB, SVM, Tree, and XGB algorithms performed significantly worse than ElasticNet with interactions.

To sum up, the investigation of the performance of the 13 algorithms revealed that ElasticNet with interactions appears to be the most practical method for small datasets. Moreover, ensemble-weighted.AUC and ensemble-weighted.F1 can be considered as the next-most-useful methods, but they are not as good as ElasticNet with interactions. In comparing linear and non-linear algorithms, the linear algorithms, including ElasticNet with and without interactions, LDA, NB, and SVM, performed better than the non-linear algorithms. Among the ensemble algorithms, those weighted by F1-score and AUC had slightly better performance.

#### 3.1.2 Benchmarks for Each Classifier in Medium-sized Datasets

We next studied the four medium-sized datasets from Packer et al. (2019); these contained fewer than 1,000 samples per label (Supplementary Table S2). The results helped us to assess how well the supervised algorithms perform with medium-sized datasets. Overall, Figure 2 confirms that the linear algorithms, in particular ElasticNet with interactions and NB, had better performance in comparison to the non-linear and ensemble algorithms.

As shown in Figure 2A, the performance of ElasticNet with interactions was still better than ElasticNet without interactions when considering AUC. In Supplementary Figure S2, it can also be seen that the stability of ElasticNet with interactions was better than the stability of the other algorithms. However, considering the F1-score and FPR criteria, the NB algorithm was the best single method, with median values of 0.994 and 0.010, respectively. The Recall of ElasticNet and NB were also considerably better than the Recall of the other algorithms. Therefore, in Figure 2B, we have marked these algorithm names under the F1-score and FPR standards.

Among the ensemble algorithms, ensemble-weighted.AUC and ensemble-addition.F1 had better performance than the other three in terms of the AUC, F1-score, Precision, and Recall criteria. These results further confirmed that the performances of ensemble-weighted.AUC and ensemble-addition.F1 were not significantly better than the best non-ensemble methods in terms of AUC and F1-score. Thus, Figure 2A reveals that ElasticNet with interactions performed the best under the AUC criterion, and NB had the best performance under the FPR and F1-score criteria. The next-most-useful methods were ensemble-weighted.AUC and ensemble-addition.F1.

As shown in Figure 2B, in terms of AUC, the *p*-value of ensemble-weighted.F1 was 0.278, that of ensemble-weighted.AUC was 0.283, and that of ElasticNet without interactions was 0.095. Thus, there were no significant differences between the AUC values of these algorithms and the AUC of ElasticNet. When the values of the F1-score and the FPR were considered, NB performed slightly better. The performance of NB was significantly better than that of all other methods, with a *p*-value of almost zero. The same results were achieved under the F1-score criterion for ensemble-weighted.F1 and ensemble-weighted.AUC, which indicates that these ensemble methods had approximately the same performance with the medium-sized datasets.

To sum up, in the medium-sized datasets, ElasticNet with interactions seems to be more practical than the other algorithms when considering the AUC criterion. The NB algorithm was the best algorithm in terms of F1-score and FPR. The ensemble-weighted.F1 and ensemble-weighted.AUC methods can also be applied as the next-most-suitable methods.

#### 3.1.3 Benchmarks for Each Classifier in Large Datasets

Next, 12 filtered datasets were applied using the datasets of Packer et al. (2019). In contrast to the medium-sized datasets, in these sets, there were over 1,000 samples of each type of cell per label, with specific values ranging from 1732 to 25,875, as described in Supplementary Table S3.

Figure 3 shows the outcomes of the performance metrics for ten algorithms (ElasticNet, LDA, NB, Tree, XGB, and five ensemble algorithms) using the large datasets; three algorithms (KNN, SVM, and ElasticNet with interactions) were set aside because of the long computation time they would require. The results revealed that XGB had the best performance when compared to the other single methods. Ensemble-weighted.F1 performed the best among the five ensemble algorithms in terms of AUC, F1-score, FPR, and Recall. In terms of stability (Supplementary Figure S3), the XGB algorithm, based on the AUC and FPR criteria, was most stable; however, ensemble-weighted.F1 had the best stability in terms of F1-score. Considering Supplementary Figure S3, the Recall of XGB was also better than that of the other algorithms. However, XGB and ensemble-weighted.F1 had the same median value of Precision.

In more detail, the results in Figure 3A show that the median value of AUC for XGB was 0.999, which was higher than that of LDA. If the parameters of ElasticNet were set the same as those used for the medium-sized and small datasets, this would cause the computation to take too long. We thus adjusted the parameters of ElasticNet in the large datasets. Overall, in Figure 3A, if the AUC, F1-score, and FPR criteria are considered, the XGB algorithm had the best performance; in contrast, XGB was not a practical method for small datasets. Furthermore, comparing the small and medium-sized datasets, the results for ElasticNet dropped from 1.000 to 0.997 to 0.993 using the AUC criterion, and the AUC for ElasticNet with the large datasets was at least 0.425. This situation caught our attention, and this will be considered further in the Discussion section. This outcome led to the selection of XGB as the best single method for use with large datasets; a comparison between this and the ensemble and other non-ensemble methods is thus displayed in Figure 3B.

As shown in Figure 3B, when considering the AUC and F1-score criteria, there were significant differences between the five ensemble algorithms and the best single method (XGB); their *p*-values were all approximately zero. However, under the FPR criterion, there were no significant differences. Thus, the other algorithms were significantly worse than XGB in terms of AUC and F1, with *p*-values less than 0.05. The single methods ElasticNet, LDA, NB, and Tree performed worse than XGB on all three measures, with *p*-values of less than 0.05 in each case.

In summary, the investigation of the performance of ten algorithms revealed that XGB is the most practical method for large datasets. Additionally, ensemble-weighted.F1 can also be used as an appropriate method for large datasets.

#### 3.1.4 Computation Time

We obtained the computation time of each algorithm for three different datasets (Table 1). For the 27 small datasets, SVM required longer to run. For the medium-sized datasets, the performance of ElasticNet, KNN, and SVM slowed notably; the computation times for these three algorithms were approximately 3.5, 24, and 10.6 times longer than the computation times for the small datasets.

By adding cross-validation to the medium-sized datasets, the SVM took a longer computation time: an average of 6,617.672 s. This result was similar to that for the small datasets. The LDA and NB algorithms required short computation times, with averages of 11.570 and 21.369 s, respectively. With the large datasets, the computation time of Tree grew little when compared to KNN.

In the measurement of each algorithm’s computation time, KNN was found to occupy too much memory with the large datasets; therefore, we could not compare the time-consuming and labor-intensive algorithms ElasticNet, KNN, and SVM. Hence, we adjusted the parameters of ElasticNet for the large datasets. For the large datasets, we calculated the computation times of five algorithms: adjusted ElasticNet, LDA, NB, Tree, and XGB (Table 1). The average computation time of XGB, as the best single method for large datasets, was 2,803.485 s.

### 3.2 Gene Selection

The dimensions of the gene variables in the scRNA-seq datasets were large. When we implemented the classification prediction, some algorithms automatically made gene selections for the datasets. Hence, we studied whether there was a particular relationship between gene-selection performance and classification-prediction performance. We examined four algorithms suitable for gene selection—NB, Tree, XGB, and ElasticNet—using the three simulated datasets provided by Soneson and Robinson (2018) (please see the Materials and Methods section for more details).

To obtain the final evaluation results, we set different randomizing seeds in each round for each dataset and performed gene selections 100 times on 100 subsets with 70*%* of the cells. Then, the results were compared with the genes in the real datasets GSE45719, GSE74596, and GSE60749–GPL13112 (Supplementary Table S4).

**FIGURE 4:**
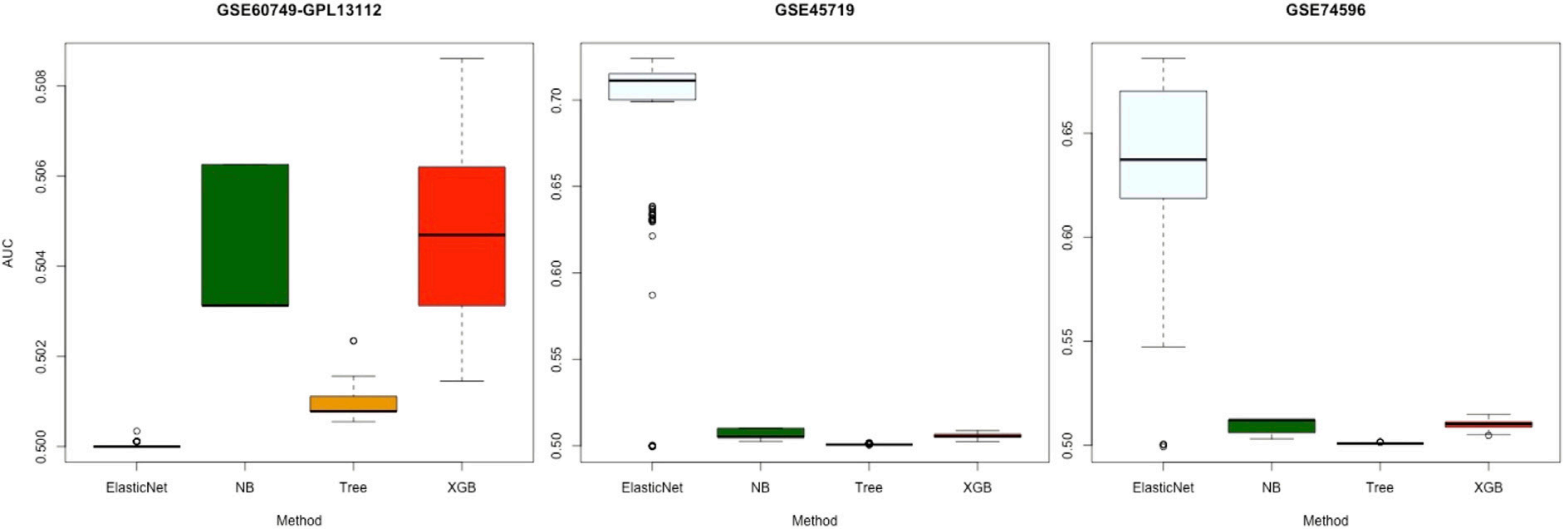
Gene-selection results from the four algorithms on 100 subsets of each simulated dataset. There are three datasets from left to right, and we have marked the dataset names at the top. Each box shows the AUC values of the four algorithms after 100 runs of gene selection on each simulated dataset. The horizontal axes are arranged by algorithm name.

Figure 4 displays the AUC values of the four algorithms—NB, Tree, XGB, and ElasticNet—for gene selection in all experiments with the three datasets. Each panel of this figure shows 100 points corresponding to the results of 100 subsets. As shown, the median value of AUC for the four algorithms on the GSE60749-GPL13112 simulated dataset was approximately 0.500. For the other two datasets, the median values for ElasticNet were about 0.711 and 0.637. This revealed that ElasticNet had better gene-selection performance than the other three algorithms.

## 4 Discussion

In this study, the classification performance and computation times of different algorithms for datasets of different sizes were evaluated and compared. A total of 27 real Conquer datasets provided by Soneson and Robinson (2018) were used for classification as small datasets, as shown in Supplementary Table S1. Four medium-sized datasets provided by Packer et al. (2019) were also used, as shown in Supplementary Table S2. The 12 large datasets provided by Packer et al. (2019) were also used for classification, as shown in Supplementary Table S3. Three simulated datasets presented by Soneson and Robinson (2018) were used to examine gene selection, as shown in Supplementary Table S4.

In this study, we conducted cross-validation experiments and gene selection, comparing the performance of various algorithms in terms of their performance, stability, computation time, and gene selection. Finally, each algorithm’s performance with different sizes of dataset was considered. According to the results, ElasticNet with interactions was the most suitable for processing small and medium-sized scRNA-seq datasets. Considering the AUC criterion, the inclusion of interactions improved the performance of ElasticNet with small and medium-sized datasets; in particular, there were significant differences between ElasticNet with and without interactions in small datasets. When ElasticNet was used with large datasets with and without interactions, there were difficulties with convergence. Hence, we adjusted the parameters, but there were problems with long computation times. Therefore, with the large datasets, we only studied ElasticNet and not ElasticNet with interactions. If the F1-score and FPR are considered, the NB algorithm can also be used as a practical approach with medium-sized datasets. The results with large datasets confirmed that if the computation time is ignored, then XGB can be regarded as the best algorithm. Additionally, this study illustrated that the ensemble algorithms were not always better than the classic linear algorithms. We also found that the integrated methods did not significantly improve the performance of the single algorithms.

### 4.1 Classification With Small Datasets

With the small datasets, the performance of the 13 algorithms was evaluated and compared using five criteria: AUC, F1-score, FPR, Precision, and Recall. Considering AUC as the standard (Figure 1), we observed that ElasticNet, ElasticNet with interactions, SVM, and LDA performed well, but LDA was unstable. The computation time of the SVM method was found to be too long to be practical. ElasticNet with interactions was a little more suitable for classification than ElasticNet without interactions in terms of performance and stability; the results showed a higher median AUC value and a lower AUC-IQR value for ElasticNet with interactions. Thus, ElasticNet with interactions can be considered as the best linear algorithm for small datasets. The performance of the XGB algorithm was better than Tree in terms of AUC and AUC-IQR. Ensemble algorithms can improve the overall generalization ability when each base learner’s performance is more significant than 0.5. Therefore, it is understandable that some kinds of ensemble strategies will perform better than others. However, we found no significantly better performance for ensemble methods compared to single methods.

The SVM algorithm performed poorly in terms of F1-score and FPR. It seems that this was because these criteria consider both performance and stability. Notably, SVM performed very slowly; this is perhaps unsurprising because the algorithm involves the selection of several optimal parameters. Both LDA and NB are simple linear classifiers with fast computation times, and their classification performances were good. ElasticNet and ElasticNet with interactions took longer than LDA; the median value of its computation time was seven times greater than that of NB but eleven times less than that of SVM. Like SVM, ElasticNet and ElasticNet with interactions also require the selection of optimal parameters to decrease the computation time. However, their computation times are practical for small datasets. Therefore, for small datasets, we suggest applying ElasticNet with interactions due to its high prediction performance and stability.

### 4.2 Classification With Medium-Sized Datasets

We compared the performance of the 13 algorithms with the medium-sized datasets. Using AUC as the standard, ElasticNet with interactions performed best, having higher performance and greater stability, as with the small datasets. The LDA and SVM algorithms did not perform well on these four datasets. The reason can be to focus on only four datasets, which may happen by chance. The XGB algorithm was also better than Tree in terms of both performance and stability. The two ensemble algorithms ensemble-weighted.AUC and ensemble-weighted.F1 also performed better; however, their performances were not significantly different from that of the single algorithm ElasticNet with interactions in terms of AUC. Considering the median value of AUC, KNN still performed more poorly than the other algorithms. We found that KNN is not suitable for processing scRNA-seq data because it requires a very large amount of memory. As the number of samples is increased, the number of calculations required by KNN also increases. Hence, in this study, there were problems with insufficient memory during these calculations.

For the F1-score and FPR standards, the NB algorithm performed better than the other algorithms. Using the AUC criterion, the performances of the other algorithms were similar. Overall, the linear algorithms continued to outperform the non-linear algorithms with medium-sized datasets. Therefore, we suggest that ElasticNet with interactions and NB are best for application with medium-sized datasets because they have better performance and stability along with moderate computation times.

### 4.3 Classification With Large Datasets

The performances of ten algorithms were compared using large datasets (Figure 3). Using AUC as the standard, the performance and computation time of ElasticNet, KNN, and SVM took longer than others. Thus, SVM and KNN were removed, and the parameters of ElasticNet were modified. We found that XGB was the best single algorithm with large datasets. ElasticNet with interactions was not considered in the final results because of its long computation time; we also found that its performance was not suitable due to some non-convergence issues. It is worth noting that ElasticNet still performed well in the convergence experiments. Therefore, ElasticNet may also be an appropriate choice after solving the convergence issues. Two of the ensemble algorithms performed as well as they did with small datasets. The core idea of the KNN algorithm is that if most of the *k* nearest samples in the feature space of a sample belong to a particular category, then the sample will also belong to that category and will have its characteristics. Hence, when the size of the datasets increases, more computation is needed (Salvador-Meneses et al., 2019). In this paper, KNN was not considered for the large datasets due to out-of-memory issues.

Overall, according to three criteria—AUC, F1-score, and FPR—the performance of the XGB algorithm was the best in most cases. We thus suggest XGB for large datasets because of its better performance and stability.

The KNN and SVM algorithms had long computation times. Therefore, they were not considered for the large datasets. The computation-time problem for ElasticNet was improved by parameter adjustment, but it was not eliminated (Table 1). The results thus illustrate that the non-convergence issue leads to more extended computation times. Moreover, the computation time of XGB is higher than other linear classifiers (LDA and NB) because it involves complicated calculations.

This study also investigated whether there was a correlation between “perfect separation” and “model non-convergence.” We collected statistics of the convergence and perfect separation in each round of 1,000 experiments for 12 large datasets. The results revealed that over 69*%* of the experiments showed “non-convergence and perfect separation” or “convergence without perfect separation.” Therefore, we propose that there is a relationship between perfect separation and model non-convergence. Moreover, considering their required computation time and performance, we suggest that LDA and XGB are more suitable for large datasets.

Finally, we discovered that the linear algorithms were better than the non-linear algorithms for classifying the different-sized datasets. Among the algorithms, ElasticNet with interactions performed well on small and medium-sized datasets. However, NB was also practical for medium-sized datasets, and XGB worked better for large datasets. There were no clear differences in the performance of the ensemble and single methods.

### 4.4 Gene Selection on Simulated Datasets

In this study, 100 subsets of three simulated datasets provided by Soneson and Robinson (2018) were used to avoid a certain level of randomness. These 100 random subsets were used with 100 experiments. The selected genes were compared with the real genes in real datasets. The results revealed that ElasticNet performed the best (Figure 4). ElasticNet achieves feature selection by regulating non-relevant predictors’ coefficients to zero (Xing et al., 2020), and this enhances the gene selection in scRNA-seq data; it is thus reasonable that it performed the best.

## 5 Conclusion

This paper presents the results of a comprehensive evaluation of supervised algorithms for the classification of scRNA-seq data to assess their performance with datasets of different sizes. The algorithms considered were as follows: 1) ElasticNet; 2) ElasticNet with interactions; 3) LDA; 4) NB; 5) SVM; 6) KNN; 7) Tree; 8) XGB; and 9) five ensemble algorithms based on the weights and votes given to the seven algorithms ElasticNet, LDA, NB, SVM, KNN, Tree, and XGB. Enormous differences were found in the performances of these algorithms in response to changing the input features. The computation times of the algorithms varied considerably with the numbers of cells and features and the type of algorithm.

According to the outcomes, ElasticNet with interactions had better performance for small and medium-sized datasets, while XGB was suitable for large datasets. The gene-selection performance of ElasticNet was perfect when comparing the genes selected by the four algorithms on the three simulated datasets with the genes in the actual datasets. It was revealed that the ensemble algorithms were not always superior to classical machine learning methods in terms of performance or computation time. The results also indicated that there were significant differences between ElasticNet with and without interactions.

We recommend ElasticNet with interactions for small and medium-sized datasets because it performs better than the other classifiers. The inclusion of interactions improved the performance of ElasticNet significantly for small datasets. The NB algorithm can also be considered as an appropriate classifier for medium-sized datasets. However, when the sample size of the datasets was large, the XGB algorithm was found to be more suitable. Although the computation time of XGB was slightly longer, its performance was relatively higher than the other methods. It seems that there is no reason to encourage the use of ensemble algorithms, as their performance was not found to be better than the performance of single methods.

## Data Availability

The original contributions presented in the study are included in the article/Supplementary Material, further inquiries can be directed to the corresponding author.
