# Supplementary material for: A Systematic Evaluation of Supervised Machine Learning Algorithms for Cell Phenotype Classification Using Single-Cell RNA Sequencing Data
Source: Front Genet. 2022 Feb 23;13:836798. doi: 10.3389/fgene.2022.836798 (PMC8905542; doi:10.3389/fgene.2022.836798)
Supplement: Supplementary file 3 [file DataSheet1.PDF]

Supplementary Material

Additional file 1: Supplementary Figures 1, 2, and 3

Supplementary Figure 1. The values of each criterion, median values, and IQR values of the AUC, F1-score, FPR, Recall, and Precision are indicated for the 27 small datasets.

Supplementary Figure 2. The values of each criterion, median values, and IQR values of the AUC, F1-score, FPR, Recall, and Precision are indicated for the four medium-sized datasets.

Supplementary Figure 3. The values of each criterion, median values, and IQR values of the AUC, F1-score, FPR, Recall, and Precision are indicated for the 12 large datasets.

Additional file 2 — Supplementary Table 1

The 27 datasets from Conquer

Additional file 3 — Supplementary Table 2

The four filtered subsets from Packer et al.

Additional file 4 — Supplementary Table 3

The 12 filtered subsets from Packer et al.

Additional file 5 — Supplementary Table 4

The 3 simulated datasets provided by Soneson et al.

0.1 Figures

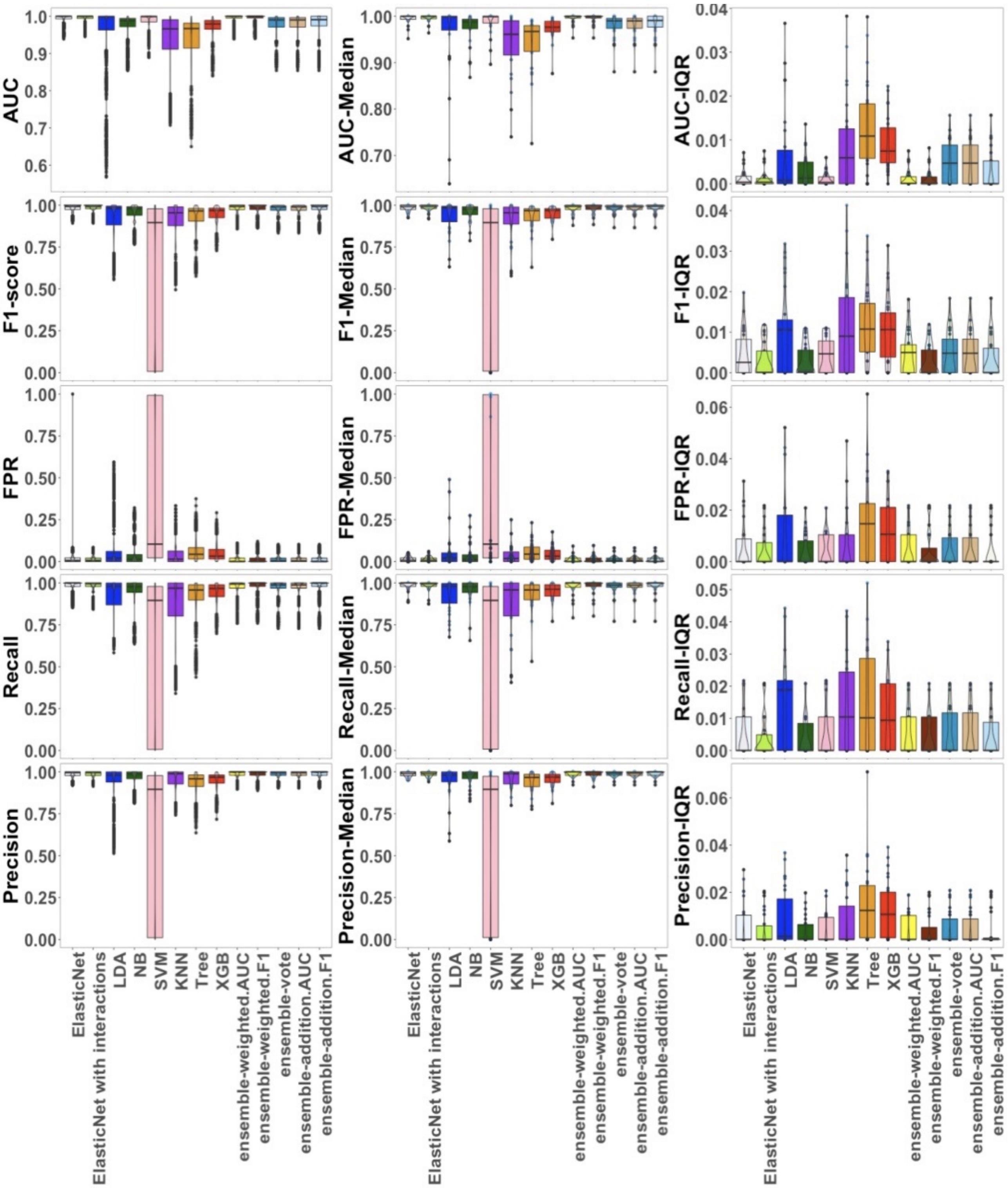

Figure S1. The values of each criterion, Median values, and IQR values of the AUC, F1-score, FPR, Recall, and Precision are indicated for 27 small data sets. The first column shows the values of AUC, F1-score, FPR, Recall, and Precision for 13 algorithms, from up to down. The median value of each criterion was used to represent the performance of the corresponding algorithm. The second column represents AUC-Median, F1-Median, FPR-Median, Recall-Median, and Precision-Median, and each box has 27 points. The third column represents AUC-IQR, F1-IQR, FPR-IQR, Recall-IQR, and Precision-IQR, from up to down. The IQR value of each criterion was applied to represent the stability of the corresponding algorithm. We have marked the criteria on the axis of each box.

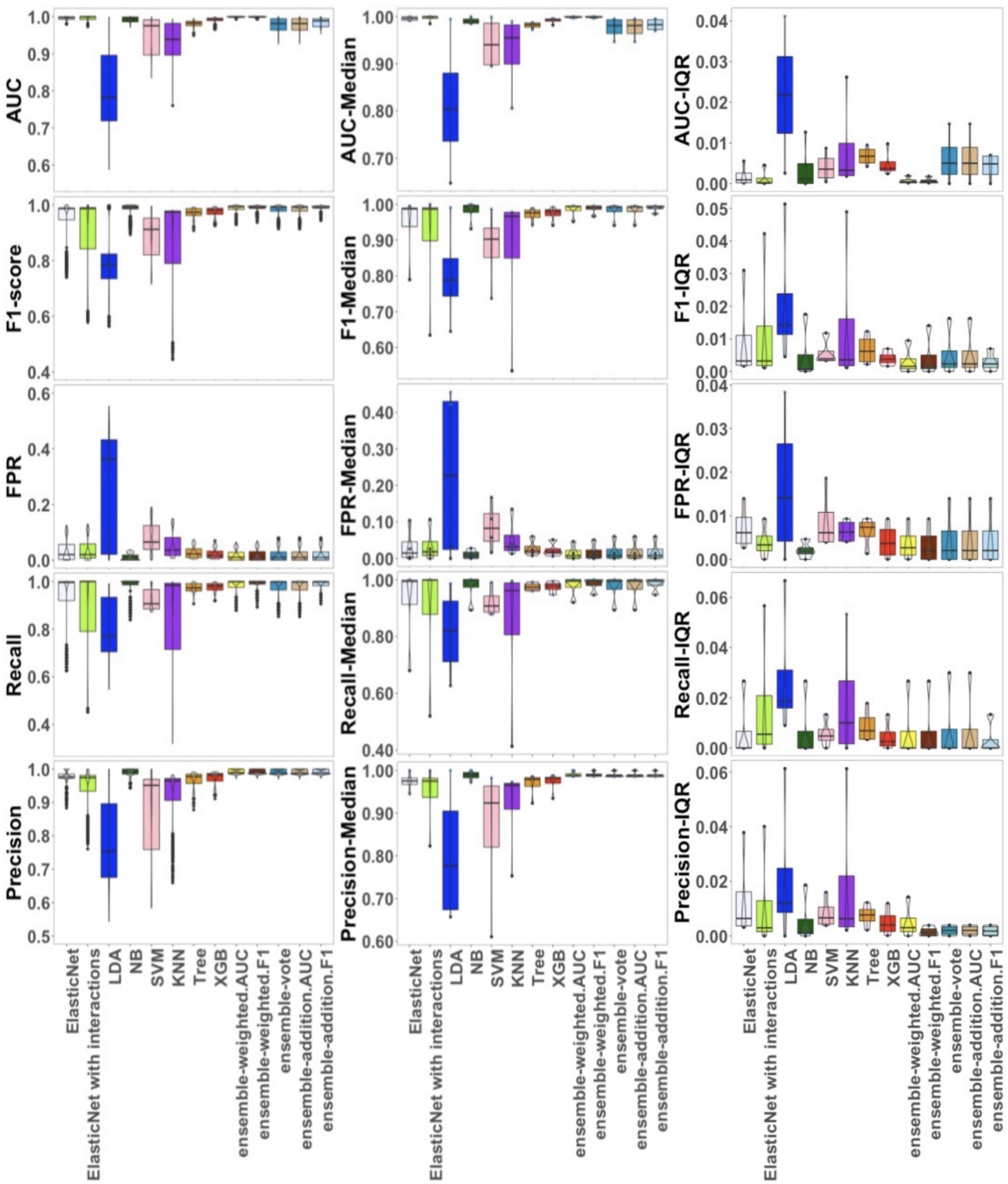

Figure S2. The values of each criterion, Median values, and IQR values of the AUC, F1-score, FPR, Recall, and Precision are indicated for 4 medium data sets. The first column shows the values of AUC, F1-score, FPR, Recall, and Precision for 13 algorithms, from up to down. The median value of each criterion was used to represent the performance of the corresponding algorithm. The second column represents AUC-Median, F1-Median, FPR-Median, Recall-Median, and Precision-Median, and each box has 4 points. The third column represents AUC-IQR, F1-IQR, FPR-IQR, Recall-IQR, and Precision-IQR, from up to down. The IQR value of each criterion was applied to represent the stability of the corresponding algorithm. We have marked the criteria on the axis of each box.

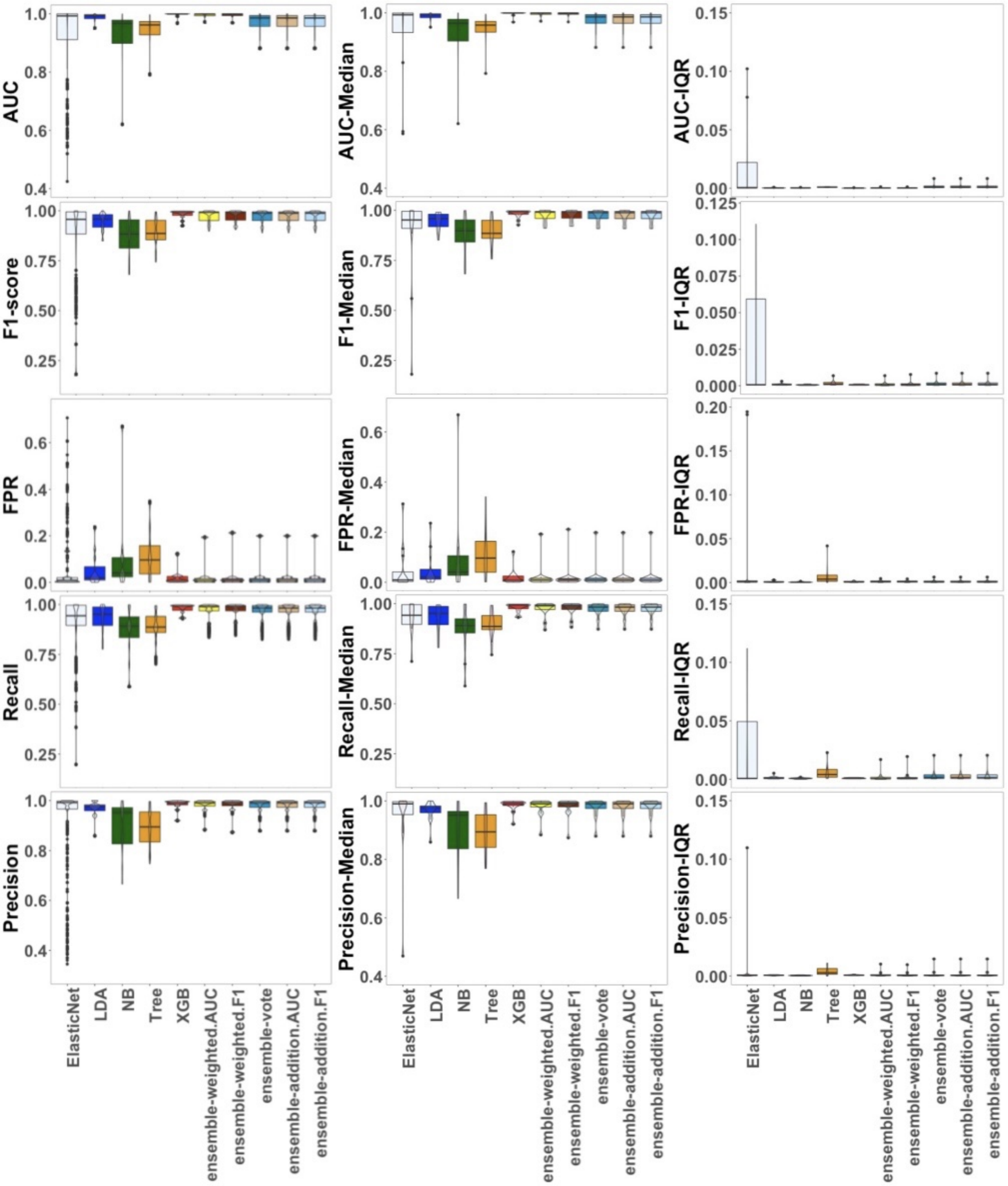

Figure S3. The values of each criterion, Median values, and IQR values of the AUC, F1-score, FPR, Recall, and Precision are indicated for 12 large data sets. The first column shows the values of AUC, F1-score, FPR, Recall, and Precision for 10 algorithms, from up to down. The second column represents AUC-Median, F1-Median, FPR-Median, Recall-Median, and Precision-Median, and each box has 10 points. The median value of each criterion was used to describe the performance of the corresponding algorithm. The third column represents AUC-IQR, F1-IQR, FPR-IQR, Recall-IQR, and Precision-IQR, from up to down. The IQR value of each criterion was applied to represent the stability of the corresponding algorithm. We have marked the criteria on the axis of each box.
